# Impact of animal and herd factors on the nonenzymatic antioxidant potential in milk from individual Brown Swiss cows

**DOI:** 10.3168/jdsc.2025-0863

**Published:** 2025-09-10

**Authors:** Irene Tedeschi, Giorgia Stocco, Michela Ablondi, Andrea Summer, Georgios Anagnostou, Alexandros Mavrommatis, Eleni Tsiplakou, Massimo Malacarne, Claudio Cipolat-Gotet

**Affiliations:** 1 Department of Veterinary Science, University of Parma, 43126 Parma, Italy; 2 Department of Animal Science, Agricultural University of Athens, 11855 Athens, Greece; 3 Food Quality and Sensory Science Department, Teagasc Food Research Centre, D15 DY05 Ashtown, Ireland

## Abstract

The objectives of this study were to (1) characterize the nonenzymatic antioxidant activity of individual bovine milk samples using 2 assays: ferric reducing antioxidant power (FRAP) and 2,2-diphenyl-1-picrylhydrazyl (DPPH), and (2) investigate the effects of animal (DIM, parity, and daily milk yield [DMY; kg/d]) and environmental factors (temperature-humidity index [THI], season, ventilation, feeding, and altitude) on these traits. Milk samples were collected once during evening milking from 1,060 Brown Swiss cows across 53 herds in Northern Italy. Antioxidant activity was expressed as micromolar ascorbic acid equivalents per milliliter of milk for the FRAP and as a percentage of DPPH inhibition. Both traits in milk were influenced by a range of animal-related and environmental factors, with varying degrees of impact. The FRAP values were highest in primiparous cows and decreased with increasing parity. In contrast, DPPH inhibition was greatest in tertiparous cows, followed by primiparous and secondiparous cows. Days in milk had a significant effect on DPPH, which declined as lactation progressed. A positive association was observed between DMY and FRAP values, with FRAP increasing up to approximately 25 kg/d, beyond which it plateaued. Among the environmental factors, the lowest FRAP values were observed in summer and in herds fed a dry TMR. Furthermore, milk from herds equipped with ventilation systems showed slightly higher DPPH inhibition compared with those without such systems. These findings offer valuable insights into factors affecting antioxidant properties in bovine milk and underscore the need for further research into other nonenzymatic antioxidants and their concentrations. Future studies should investigate the genetic basis of milk antioxidant properties, examine their relationship with milk composition, and evaluate the consequences for animal health and product quality.

## Highlights

• Antioxidant activity in milk is affected by both animal-specific and environmental factors.
• Distinct patterns were observed between FRAP and DPPH assays.
• Environmental conditions significantly modulate milk antioxidant activity.

## Graphical Abstract

**Figure:**
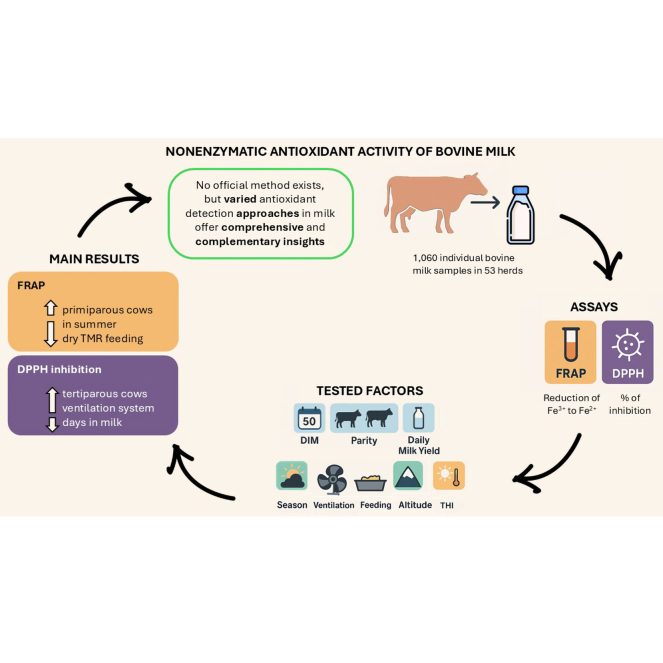
**Summary:** This study uncovered key findings on the antioxidant activity of individual bovine milk samples based on data from 1,060 cows across 53 herds. Antioxidant capacity, measured as ferric reducing antioxidant power (FRAP) and expressed in micromolar ascorbic acid equivalents per milliliter of milk, was found to progressively increase over the course of lactation, with increasing parity and during summer. Variations in antioxidant activity were also observed based on the type of feeding regimen. The FRAP increased progressively from dry total mixed ration (TMR), to conventional feeding, and then to standard TMR, possibly reflecting differences in forage intake and botanical composition of the diet. Additionally, the 2,2-diphenyl-1-picrylhydrazyl (DPPH) radical scavenging activity, expressed as percentage inhibition, decreased over the course of lactation, and increased with parity and the presence of ventilation. These factors appear to affect specific milk components that act as radical scavengers.

Bovine milk is a rich source of lipophilic (e.g., CLA, α-tocopherol, β-carotene) and hydrophilic (e.g., ascorbate, phenols) antioxidants that mitigate oxidative stress by neutralizing reactive oxygen species (Grażyna et al., 2017). Bioactive peptides from casein and whey also exhibit antioxidant properties. Milk antioxidant capacity is influenced by multiple factors, including composition, animal physiology, feeding, environmental conditions, and the analytical method used (Santa et al., 2022). Due to the complexity of milk as a matrix, no single in vitro assay can comprehensively assess its antioxidant capacity. The ferric reducing antioxidant power (**FRAP**) assay measures the reduction of ferric to ferrous ions in acidic conditions (Benzie and Devaki, 2018), whereas the 2,2-diphenyl-1-picrylhydrazyl (**DPPH**) assay quantifies the scavenging of stable free radicals via hydrogen or electron donation (Nenadis and Tsimidou, 2018). Despite methodological limitations, evaluating nonenzymatic antioxidants, especially those linked to milk composition, offers valuable insights into the oxidative stability of milk. Moreover, understanding how animal- (e.g., lactation stage, parity) and herd-level factors (e.g., feeding, ventilation) affect antioxidant variability is crucial for optimizing dairy practices and enhancing the health-promoting potential of milk. Therefore, the aim of this study was to analyze animal-related factors (DIM, parity, daily milk yield [**DMY**; kg/d]), and environmental conditions (ventilation, feeding, season) on the variability of nonenzymatic antioxidant activity in individual bovine milk samples. This study is part of the GENEtoCHEESE project (number D94I19000840001) funded by the Italian Ministry of Agricultural, Food and Forestry Policies (MiPAAF, Rome, Italy). A total of 1,060 Brown Swiss cows were sampled across 53 dairy herds in Northern Italy. Each herd was visited once during evening milking, and 20 cows per herd were sampled at each visit. Cows were also characterized by DIM, parity, and DMY. Herds were characterized by the presence (n = 46) or absence (n = 8) of ventilation. Environmental data, including minimum and maximum temperatures (−3°C to 33°C) and relative humidity (25%–100%), were obtained from the nearest meteorological stations. These values were used to calculate the temperature-humidity index (**THI**) following the equation described by Mader et al. (2006). These THI values were then grouped into 3 categories: <73 (n = 38 herds), 73–79 (n = 8 herds), and 80–84 (n = 7 herds). Herds were also categorized into 3 feeding systems: TMR (n = 9 herds), consisting of long forage, concentrates, and mixed byproducts; dry TMR (n = 27 herds), in which water is added during feed preparation; and conventional feeding (n = 17 herds), where individual feed components are offered separately. Altitude of the herds was classified as plains (n = 36), hills (n = 10), or mountains (n = 7).

Nonenzymatic antioxidant activity was determined using the FRAP and DPPH assays, as described by Tsiplakou et al. (2018). The FRAP was expressed as micromolar equivalents of ascorbic acid per milliliter of milk, and the DPPH activity was expressed as percentage inhibition.

The milk FRAP and DPPH traits were analyzed using R Studio software (R Core Team, 2023) according to the following linear mixed model:

*y_mnopqrstuv_* = *μ* + *THI_n_* + *Season_o_* + *Ventilation_p_* + *Feeding_q_* + *Altitude_r_* + *Sampling day_s_*(*THI* × *Season* × *Ventilation* × *Feeding* × *Altitude*)*_nopqr_* + *DIM_t_* + *Parity_u_* + *DMY_v_* + *e_mnopqrstuv_*,

where *y_mnopqrstuv_* is the *m*th nonenzymatic antioxidant activity (FRAP and DPPH traits); *μ* is the overall intercept of the model; *THI_n,_* is the fixed effect of the *n*th class of THI (*n* = 1 to 3 classes of THI; class 1: <72 [760 samples]; class 2: 73–79 [160 samples]; class 3: 80–84 [140 samples]); *Season_o_* is the fixed effect of the *o*th season [*o* = winter (160 samples), spring (360 samples), summer (220 samples), autumn (320 samples)]; *Ventilation_p_* is the fixed effect of the *p*th class of ventilation (*p* = presence [900 samples] or absence [160 samples]); *Feeding_q_* is the fixed effect of the *q*th class of feeding type (*q* = 1 to 3 classes of diet; class 1: conventional [340 samples]; class 2: dry TMR [540 samples]; class 3: TMR [180 samples]); *Altitude_r_* is the fixed effect of the *r*th class of altitude (*r* = 1 to 3 classes; class 1: plains [740 samples]; class 2: hills [200 samples]; class 3: mountains [120 samples]); *Sampling day_s_* is the uncorrelated random effect of the *s*th herd sampling day (*s* = 1 to 53) within the *n*th *THI*, *o*th *Season*, *p*th *Ventilation*, *q*th *Feeding*, and *r*th *Altitude*; *DIM_t_* is the fixed effect of the *t*th class of DIM (*t* = 1 to 6; class 1: <60 d [132 samples]; class 2: 61–120 d [211 samples]; class 3: 121–180 d [200 samples]; class 4: 181–240 d [153 samples]; class 5: 241–300 d [150 samples]; class 6: >300 [214 samples]); *Parity_u_* is the fixed effect of the *u*th parity (*u* = 1 to 5; class 1: first [293 samples]; class 2: second [263 samples]; class 3: third [245 samples]; class 4: fourth [147 samples]; class 5: ≥fifth [112 samples]); *DMY_v_* is the fixed effect of the *v*th DMY (*v* = 1 to 5 classes of quintiles; class 1: [225 samples]; class 2: [203 samples]; class 3: [218 samples]; class 4 [214 samples]; class 5: [186 samples]); and *e_mnopqrstuv_* is the residual random error term ∼*N*(0, *σ*^2^). Orthogonal contrasts were estimated among the LSM for THI (<72 THI vs. others, and 73–79 THI vs. 80–84 THI), altitude (hills vs. plains, mountains vs. plains), season (winter vs. summer, summer vs. autumn, spring vs. summer), feeding (conventional vs. dry TMR, conventional vs. TMR silage), and parity (first vs. ≥second, second vs. ≥third, and third vs. ≥fourth parity). For the DIM and the DMY effects polynomial contrasts were estimated (linear, quadratic, and cubic trend). For data editing and preprocessing, the *dplyr* (Wickham et al., 2023), *rstatix*, *dplyr*, *lubridate*, and *tidyverse* packages (Wickham et al., 2019) were used. For the linear mixed model, the *lme4*, *lmer*, and *emmeans* packages (Gilbert, 2024) were used.

In scientific literature, the nonenzymatic antioxidant activity in raw cow milk is commonly measured using FRAP and DPPH assays, though results are reported in various units, such as micromoles of Trolox equivalents (**TE**)/L, milligrams of TE, or percent inhibition, complicating comparisons across studies (Olszowy-Tomczyk, 2021). For instance, Amamcharla and Metzger (2014) analyzed 647 raw milk samples using FRAP, testing different treatments including copper sulfate, vitamin E, and their combination, with results ranging from 81.1 to 460.4 mmol/L FeSO_4_·7H_2_O. Yilmaz-Ersan et al. (2018) found average antioxidant values of 1.41 mg of TE (FRAP) and 3.14 mg of TE (DPPH) in bovine milk. In the present study, the mean value of FRAP was 2.20 μ*M* equivalents of ascorbic acid per milliliter of milk, whereas the DPPH assay revealed a mean radical scavenging activity of 59.7% inhibition (Table 1). The high average DPPH inhibition, coupled with its low variability (CV = 4%), may point to a tightly conserved pool of hydrogen-donating antioxidants, possibly related to lipophilic compounds or to innate milk defense mechanisms (e.g., lactoferrin, tocopherols). The variability between the 2 assays may reflect differences in the antioxidant compounds they detect. The FRAP measures water-soluble reducing agents, which can vary with diet, health, and metabolism, whereas DPPH targets hydrogen or electron donors, which may be more tightly regulated in milk. These findings align with Stocco et al. (2024), who assessed nonenzymatic antioxidant activity in sheep milk using the same methods. They reported an average FRAP value of 2.24 μ*M* ascorbic acid equivalents/mL (±0.54 SD) and a mean DPPH inhibition of 63.4% (±2.29% SD). The low phenotypic correlation between FRAP and DPPH (r = 0.17) further supports that the assays measure different antioxidant components in milk. Table 1 also presents the ANOVA results. The FRAP values were significantly affected by parity (*P* = 0.027) and DMY (*P* < 0.001), as well as by season (*P* = 0.001) and feeding system (*P* = 0.072), underscoring the multifactorial influences on milk antioxidant capacity. Figure 1a shows that primiparous cows had the highest FRAP values (2.31 μ*M* ascorbic acid equivalents/mL), followed by secondiparous, tertiparous, and cows with over 4 parities. This trend may reflect parity-related differences in iron-binding proteins. Primiparous cows have usually lower milk transferrin but higher transferrin saturation index (**TSI**), meaning fewer transferrin molecules carry more iron (Moretti et al., 2020). This increases redox-active iron and FRAP values, suggesting a physiological adaptation to enhance neonatal iron uptake and immunity. With increasing parity, transferrin and total iron-binding capacity rise, TSI decreases, and FRAP values decline, linking the FRAP gradient to iron saturation in milk. A positive relationship between DMY and FRAP was observed (Figure 1b), with values rising from 4.22 to 4.62 μ*M* ascorbic acid equivalents/mL as DMY increased from 15.04 to 36.98 kg/d. The pattern was linear up to 25 kg/d, then plateaued, suggesting a physiological limit to antioxidant increase in high-producing cows, while low-producing cows may have a more efficient antioxidant response. Niero et al. (2020) reported a negative association (r = −0.184) between milk yield and total antioxidant activity (**TAA**) in milk, indicating that high-producing cows may exhibit lower TAA, supporting the hypothesis of dilution effect. Nevertheless, it is important to note that TAA encompasses a broader spectrum of antioxidant molecules than those measured by FRAP and DPPH assays, which may explain the discrepancies between our results and previous studies. Du et al. (2024) reported that high-producing cows had lower aspartate aminotransferase and bilirubin levels, but higher serum antioxidant capacity than low-producing cows, indicating better liver health. This may help sustain milk production and oxidative stress management, partially explaining the FRAP levels observed in the milk of high-producing cows in our study. Seasonal differences in FRAP values were evident (Figure 1c), with the lowest in summer (2.13 μ*M*) and highest in autumn (2.46 μ*M*). Although literature on individual-level seasonal variation is limited, factors like forage quality, feed composition, climate, lactation stage, and animal health may contribute (Marino et al., 2012). The FRAP values followed a seasonal pattern similar to casein and fat content (data not shown), possibly reflecting cows' physiological responses to temperature changes (Liu et al., 2019). In summer, heat stress reduces feed intake, affecting milk yield and composition, including fat, protein, vitamins, and iron-reducing compounds. The seasonal effect, which includes THI variations, likely results from multiple interacting factors influencing antioxidant activity throughout the year. Regarding feeding type (Figure 1d), the highest FRAP value was found in milk from cows fed silage TMR (2.35 μ*M*), followed by the conventional diet (2.24 μ*M*), with the lowest in cows on dry TMR (2.14 μ*M*). Although TMR systems provide a balanced ration, cows may sort feed, favoring fine particles high in degradable carbohydrates and low in fiber, risking ruminal acidosis. Moistening dry TMR can reduce sorting (Shaver, 2002) but may also decrease intake due to faster fermentation and reduced palatability (Miller-Cushon and DeVries, 2009). Lower intake on dry TMR may reduce nutrients needed for antioxidant synthesis measured by FRAP, despite tailored supplementation. Supporting this, De La Torre-Santos et al. (2021) found higher α-tocopherol, β-carotene, and retinol in milk from cows on supplemented TMR diets, both known antioxidants. Conventional feeding is more variable due to hay composition, season, and supplementation limits, especially in Protected Designation of Origin systems, explaining some differences (Marino et al., 2012). For DPPH, DIM (*P* = 0.022) and parity (*P* = 0.031) were key animal-related factors, whereas ventilation had a marginal effect (*P* = 0.081) among environmental factors. Figure 2a shows that antioxidant activity measured by DPPH decreased notably with increasing DIM. This decline likely reflects rising oxidative stress during lactation due to higher metabolic demands, body reserve mobilization, antioxidant depletion, changes in milk composition, and possible nutritional deficits. Indeed, it is important to emphasize that these outcomes arise from complex interactions among multiple metabolic and nutritional factors. Therefore, further studies are warranted to elucidate the underlying mechanisms and confirm these associations. Regarding the effect of parity (Figure 2b), DPPH inhibition was highest in milk from tertiparous cows (59.89%), followed by secondiparous (59.52%) and primiparous cows (59.62%). When grouped more broadly, multiparous cows showed an average value of 59.83% of inhibition. These findings indicate a comparatively lower antioxidant capacity in the milk of second-parity cows. Moreover, higher DPPH values in older cows suggest increased oxidative status in their milk, likely due to age-related oxidative damage, reduced antioxidant defenses, and metabolic changes that raise oxidative stress. Supporting this interpretation, Morales Piñeyrúa et al. (2018) found that multiparous cows produce milk with higher fat content (4.2%) than primiparous cows (3.7%), which may increase milk susceptibility to oxidation. This, along with age-related hormonal and physiological changes, could explain the higher oxidative status seen in milk from older cows. Additionally, Pokorska et al. (2019) studied lipocalin-2 gene polymorphisms and their link to milk antioxidant capacity (measured by TE). One polymorphism was linked to higher antioxidant levels, varying with cow age and parity. Milk from older cows (>6 yr) showed considerably higher antioxidant activity (4.07 µ*M* TE) than younger cows (≤4 yr, 1.25 µ*M* TE), likely due to increased free radical production with age, triggering greater antioxidant defense (Kirkwood and Kowald, 2012). Regarding the effect of ventilation (Figure 2c), milk produced from herds with ventilation showed 59.99% inhibition compared with 59.44% in those without ventilation. Although no specific study has investigated the effect of herd characteristics on the antioxidant activity of bovine milk, Broucek et al. (2020) analyzed 2 herds with different cooling systems. One herd employed evaporative cooling system, whereas the other used only forced ventilation. The study showed that cows cooled by evaporation produced more milk, although the fat and protein content was higher in cows reared in forced ventilation housing, indicating a dilution effect.

**Table 1:** Descriptive statistics (mean ± SD), ANOVA (*F*-value and significance), and results of contrasts (contrast coefficient and significance) for FRAP and DPPH measured in individual bovine milk samples^1^

Item	FRAP, μ*M* ascorbic acid equivalents/mL of milk	DPPH, % inhibition
Mean ± SD	2.2 ± 0.48	59.7 ± 2.6
CV, %	22	4
Tested factor		
DIM	1.69	2.32*
Linear	0.18	−2.94*
Quadratic	0.16	0.11
Cubic	0.89	1.84
Parity	2.91*	3.15*
First vs. ≥second	0.09**	−0.13
Second vs. ≥third	0.02	−0.35**
Third vs. ≥fourth	0.02	0.07
Daily milk yield	5.32***	0.31
Linear	0.46***	0.18
Quadratic	−0.25*	−0.07
Cubic	0.10	0.35
Temperature-humidity index	0.19	1.07
<72 vs. others	0.09	0.40
73–79 vs. 80–84	0.02	0.34
Altitude	2.02	1.41
Plains vs. mountains	−0.01	−0.30
Plains vs. hills	0.19	−0.37
Hills vs. mountains	0.20	−0.08
Season	5.37**	1.76
Winter vs. summer	−0.04	0.91
Summer vs. autumn	−0.13	−0.5
Spring vs. summer	−0.56	0.98
Feeding type	2.52†	1.94
Conventional vs. TMR silage	−0.11	−0.51
Conventional vs. dry TMR	−0.09	0.19
Dry TMR vs. TMR silage	−0.20	−0.32
Ventilation	0.81	4.05†
No vs. yes	0.10	0.54†

1 FRAP = ferric reducing antioxidant power; DPPH = 2,2-diphenyl-1-picryl-hydrazyl-hydrate.

*** *P* < 0.001

** *P* < 0.01,

* *P* < 0.05; †*P* < 0.1.

**Figure 1:**
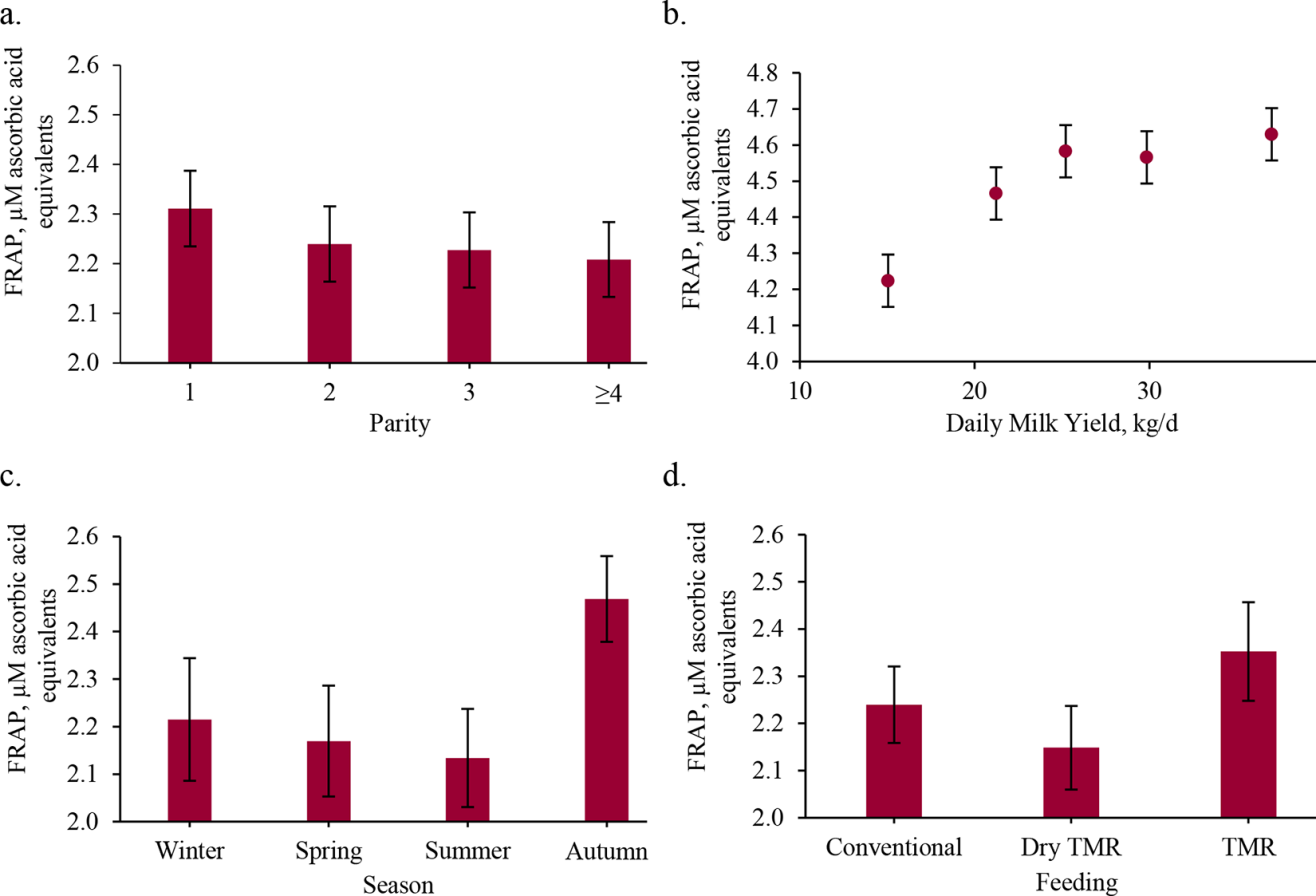
Effect of parity (a), daily milk yield (b), season (c), and feeding type (d) on FRAP of bovine milk samples. Errors bars represent SE of the LSM.

**Figure 2:**
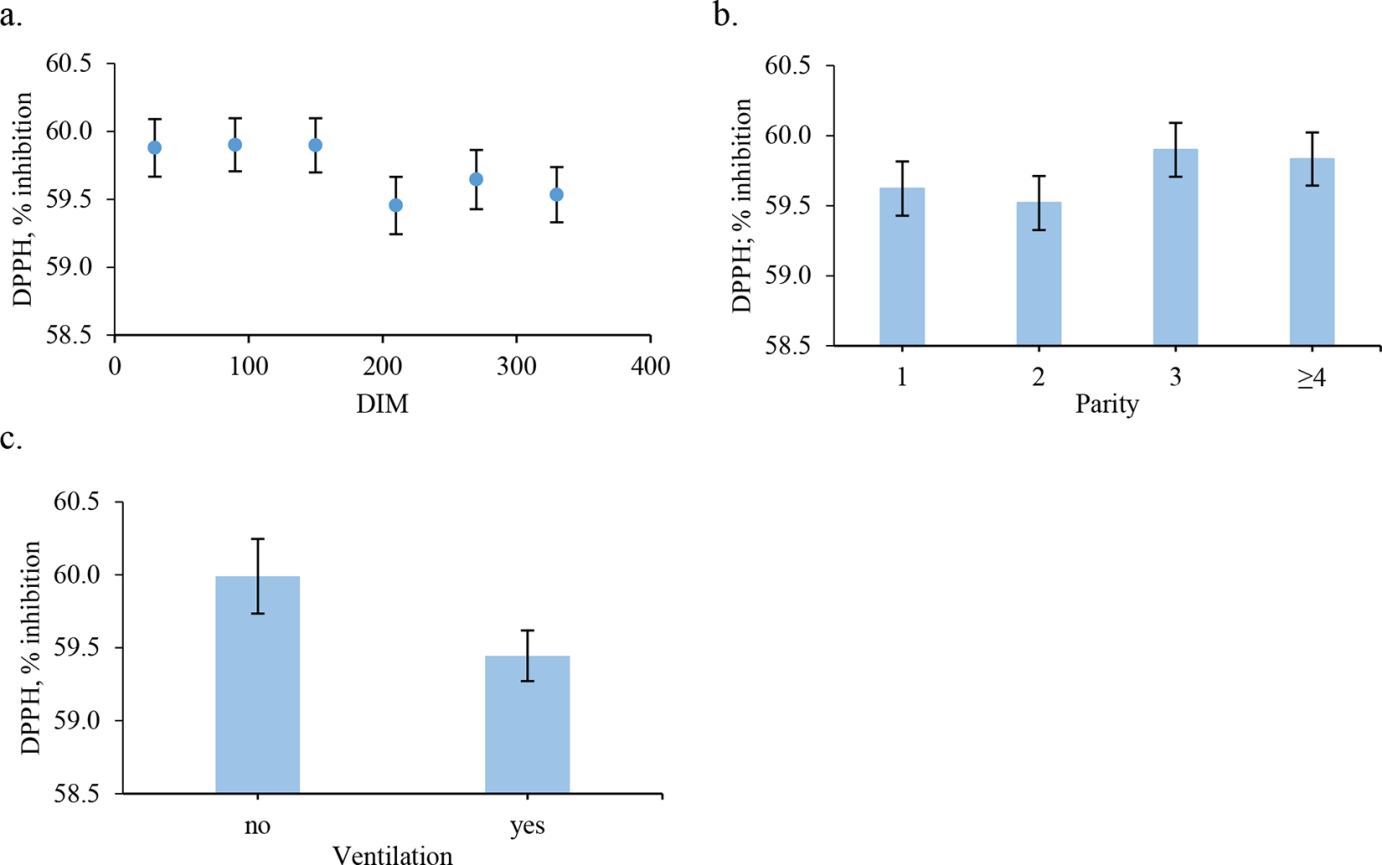
Effect of DIM (a), parity (b), and ventilation (c) on the DPPH of bovine milk samples. Errors bars represent SE of the LSM.

These results underscore the multifactorial nature of antioxidant activity in bovine milk, influenced by both animal and environmental factors. The FRAP and DPPH responded differently, suggesting distinct mechanisms. Further research should examine how antioxidant capacity relates to milk's nutritional and technological quality and explore the genetic basis of these traits and their link to milk composition. Such efforts could have broader implications, contributing to improved animal welfare, enhanced product quality, and the development of more sustainable and health-oriented dairy systems.
